# Genetic polymorphisms of methylenetetrahydrofolate reductase C677T and risk of ischemic stroke in a southern Chinese Hakka population

**DOI:** 10.1097/MD.0000000000013645

**Published:** 2018-12-21

**Authors:** Jingyuan Hou, Xing Zeng, Yunquan Xie, Hesen Wu, Pingsen Zhao

**Affiliations:** a Clinical Core Laboratory; b Center for Precision Medicine, Meizhou People's Hospital (Huangtang Hospital), Meizhou Academy of Medical Sciences, Meizhou Hospital Affiliated to Sun Yat-sen University; c Guangdong Provincial Engineering and Technology Research Center for Molecular Diagnostics of Cardiovascular Diseases; d Meizhou Municipal Engineering and Technology Research Center for Molecular Diagnostics of Cardiovascular Diseases; e Meizhou Municipal Engineering and Technology Research Center for Molecular Diagnostics of Major Genetic Disorders; f Prenatal Diagnosis Center, Meizhou People's Hospital (Huangtang Hospital), Meizhou Academy of Medical Sciences, Meizhou Hospital Affiliated to Sun Yat-sen University; g Guangdong Provincial Key Laboratory of Precision Medicine and Clinical Translational Research of Hakka Population, Meizhou, P.R. China.

**Keywords:** C677T, Hakka population, ischemic stroke, methylenetetrahydrofolate reductase gene, polymorphisms

## Abstract

Previous studies have shown that methylenetetrahydrofolate reductase (*MTHFR*) gene to be a genetic risk factor for the susceptibility to ischemic stroke. The aim of this case-control study was to investigate whether the polymorphisms of *MTHFR* C677T were associated with the susceptibility to ischemic stroke in a southern Chinese Hakka population. In this study, a total of 1967 ischemic stroke patients and 2565 controls of Chinese Hakka ethnicity were recruited. The *MTHFR* C677T polymorphisms were genotyped by polymerase chain reaction (PCR) amplification and microarray method. The risk of ischemic stroke was estimated by logistic regression analysis. The frequencies of CC, CT, and TT genotypes were 52.67% versus 55.63%, 40.31% versus 38.52%, and 7.02% versus 5.85% in patients with ischemic stroke versus controls, respectively. The frequency of T allele was significantly higher in ischemic stroke patients (27.17%) than in controls (25.11%) (*P* = .026, odds ratio [OR] 1.113, 95% confidence interval [CI] 1.013–1.223). The homozygous TT genotype in the ischemic stroke patients was associated with increased risk (*P* = .049, OR 1.132, 95% CI 1.001–1.281) when compared with the controls after adjustment for age and sex. The positive association was only found in dominant model without adjustment for age and sex (*P* = .047, OR 1.127, 95% CI 1.002–1.268). Also, the carrier status of the *MTHFR* T allele was identified as an independent risk factor for the development ischemic stroke even after the adjustment for conventional risk factors (*P* = 0.047, OR 1.109, 95% CI 0.964–1.225). Our results provide evidence that variants of *MTHFR* C677T gene may influence the risk of developing ischemic stroke in a southern Chinese Hakka population. Further studies are needed to confirm this association, which will promote the development of strategies for prevention and treatment of ischemic stroke in our study population.

## 1 Introduction

Stroke is the second leading cause of death globally and a prominent cause of reduced disability-adjusted life-years worldwide.^[1,2]^ Epidemiological studies have reported that there are 2.5 million new cases of stroke, and more than one million people suffered from stroke-related causes every year in China.^[3]^ Ischemic stroke is the most common type of stroke that accounts for more than 80% of all stroke events, and it occurs when an artery that supplies blood to the brain is interrupted. The etiology of ischemic stroke is heterogeneous and widely accepted as a complex multifactorial disease influenced by interactions between genetic and environmental factors.^[4–6]^ Proper management of traditional risk factors for ischemic stroke, such as hypertension, diabetes mellitus, hypercholesteremia, cigarette smoking, excessive drinking, and heart diseases, may reduce the incidence of ischemic stroke only to a certain degree.^[7–9]^ To date, extensive research has been done focusing on the relationship between genetic variants and susceptibility to ischemic stroke.

Methylenetetrahydrofolate reductase gene (*MTHFR*) maps to chromosomal location 1p36.3, which is involved in the amino acid and purine biosynthesis pathway.^[10,11]^*MTHFR* is a critical regulatory enzyme that catalyzes the transformation of 5,10-methylenetetrahydrofolate to 5-methyltetrahydrofolate, which serves as the methyl group donor needed for the conversion of homocysteine to methionine.^[12,13]^ A common mutation for the C to T substitution at nucleotide 677 of *MTHFR* gene (rs1801133, C667T), which is associated with decreased enzyme activity and eventually leads to elevation of plasma homocysteine levels.^[14,15]^ Hyperhomocysteinemia has been reported to be associated with a variety of metabolic disorders and increased risk for complex diseases, including stroke.^[16–19]^ Despite the *MTHFR* C677T polymorphisms is recognized as a risk factor for ischemic stroke, there are no consistent results among the populations studies.^[16,20–22]^ These conflicted results may be due to small sample size, various ethnic groups, and lack of consideration of lifestyle. Additionally, to our knowledge, no previous report has examined the effect of *MTHFR* C677T polymorphisms on the risk of ischemic stroke in a Hakka population. Therefore, the aim of this case-control study was to investigate whether the polymorphisms in *MTHFR* C677T gene were associated with the susceptibility to ischemic stroke in a southern Chinese Hakka population.

## 2 Methods

### 2.1 Study population

**Figure 1:**
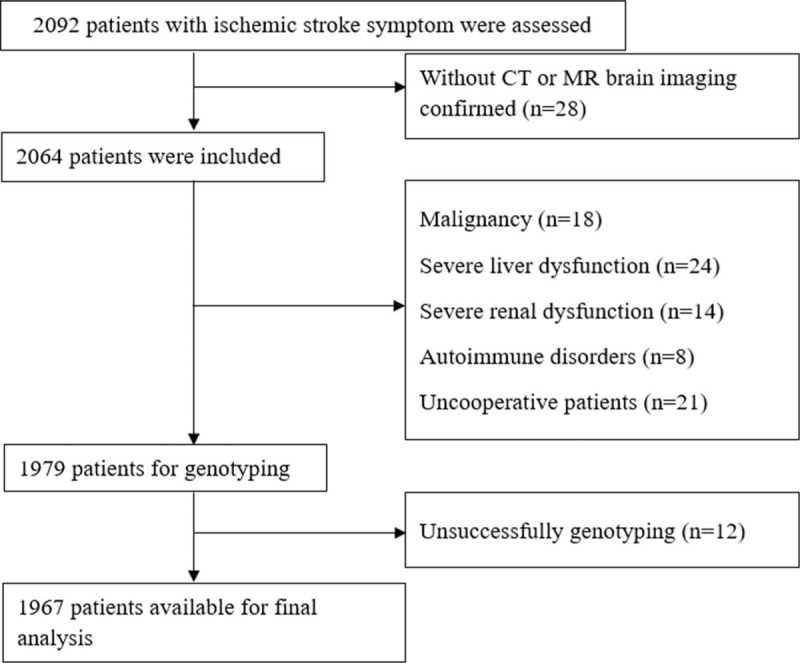
Study flow chart showing the number of patients included in the final analysis.

Meizhou is a small town located in the northeast of Guangdong Province, with a total area of 15,876 m^2^ and a population of 5.43 million, and approximately 95% inhabitants in Meizhou are Hakka. In this retrospective case-control study, 1967 consecutive unrelated subjects (1293 males and 674 females) were recruited from the patients who visited the neurology department of Meizhou People's Hospital (Huangtang Hospital), Meizhou Academy of Medical Sciences, Meizhou Hospital Affiliated to Sun Yat-sen University, Guangdong Province, China, from January 2015 to March 2018. All subjects were newly diagnosed as ischemic stroke and confirmed by strict neuroimaging with computed tomography (CT) or magnetic resonance (MR) brain imaging. Patients were excluded if they have proven intracerebral bleed or other concomitant serious medical illness such as malignancy, severe hepatic and renal dysfunction, malignant anemia, and autoimmune disorders. Two thousand and five hundred sixty-five unrelated healthy control subjects were consecutively selected with an absence of personal history of stroke (1565 males and 1000 females). Study flow diagram of patients’ enrollment was shown in Fig. 1. The study protocol was adhered to the Declaration of Helsinki and was ethically approved by the Ethics Committee of Meizhou People's Hospital (Huangtang Hospital), Meizhou Academy of Medical Sciences, Meizhou Hospital Affiliated to Sun Yat-sen University, Guangdong Province, China. All participants in this study provided signed informed consent before the study.

Specially trained interviewers administered a standardized questionnaire. A careful anamnestic evaluation and physical examination were performed in all participants. All patients underwent a comprehensive medical history, physical examination, and clinical biochemistry analysis at the time of ischemic stroke. Risk factors for ischemic stroke were recorded, including age, sex, history of hypertension, diabetes mellitus, smoking, drinking alcohol, and dyslipidemia.

### 2.2 MTHFR genotyping

A 5-mL peripheral venous blood sample was drawn and collected in vacuum tubes containing ethylene diamine tetra acetic acid (EDTA). Genomic DNA was isolated from the blood by using a commercial DNA isolation kit (Qiagen, Hilden, Germany) according to the manufacturer's protocol. All DNA samples were stored at −80°C until use. Genotyping of *MTHFR* C677T (rs1801133) polymorphisms was performed by polymerase chain reaction (PCR) amplification and microarray method using a commercially available kit (BaiO Technology Co, Ltd, Shanghai, China). The 25-μL PCR reaction mixture contained 20 ng DNA template and the recommended amounts of primers, dNTPs, and Taq DNA polymerase. The PCR condition was as follows: predenaturing at 94°C for 5 minutes, then 35 cycles (94°C for 25 seconds, 56°C for 25 seconds, and 72°C for 25 seconds), and final extension at 72°C for 5 minutes. The PCR products were then dispensed into a hybridization reaction chamber for hybridize reactions. Genotypes of *MTHFR* C677T were visualized by using the BaiO Array Doctor Version2.0 software and BaiOBE-2.0 software according to the instructions of the manufacturer (BaiO Technology Co, Ltd, Shanghai, China). The genotyping results were confirmed by direct sequencing of the PCR product with an ABI 3500xL DNA sequence analyzer by using a commercially available kit (SinoMDgene Technology Co., Ltd., Beijing, China).

### 2.3 Statistical analysis

Statistical analyses were performed using Statistical Package for the Social Sciences (SPSS) version 20.0 statistics software (SPSS Inc., Chicago, IL). Continuous data are expressed as means ± standard deviation and categorical data are expressed as percentages. The significance of differences between continuous variables was determined by Student *t* test or 1-way analysis of variance (ANOVA). Differences between categorical variables were evaluated with a chi-square test. Allele frequency was determined via direct counting, and the standard goodness-of-fit test was used for the testing of Handy–Weinberg equilibrium (HWE) by chi-square test. Univariate and multivariate logistic regressions were used to estimate odds ratio (OR) and 95% confidence interval (CI) for the association between ischemic stroke and any of the confounds. Differences were significant at *P* < .05.

## 3 Results

### 3.1 Baseline characteristics

A total of 1967 ischemic stroke patients and 2565 controls who met our inclusion criteria were enrolled in this study. The general baseline clinical characteristics are present in Table 1. Analysis of baseline clinical characteristics of the 2 groups showed that there were significant differences in the mean age for which 68.6 ± 12.1 years for the patients and 65.1 ± 13.2 years for the controls. Both systolic blood pressure (SBP) and diastolic blood pressure (DBP) were significantly higher in the ischemic stroke patients compared with the controls. As expected, the ischemic stroke patients had a significantly higher prevalence of smoking, hypertension, diabetes, dyslipidemia, and hyperhomocysteinemia (*P* < .05). There were no significant differences in the rate of drinking between the groups (*P* = .511). Moreover, the high-density lipoprotein (HDL) cholesterol level was lower (*P* < .001) and total plasma homocysteine level was significantly higher in the patient group than in the control group (*P* < .001). However, there was no significant difference in total cholesterol, triglycerides, and low-density lipoprotein (LDL) cholesterol levels between the 2 groups (*P* > .05). Table 2 shows the baseline clinical characteristics of ischemic stroke patients according to *MTHFR* C677T genotypes. The prevalence of hyperhomocysteinemia (*P* = .004) and the level of homocysteine (*P* < .001) were significantly higher in the TT genotype group than in the CC and CT genotype groups.

**Table 1:**
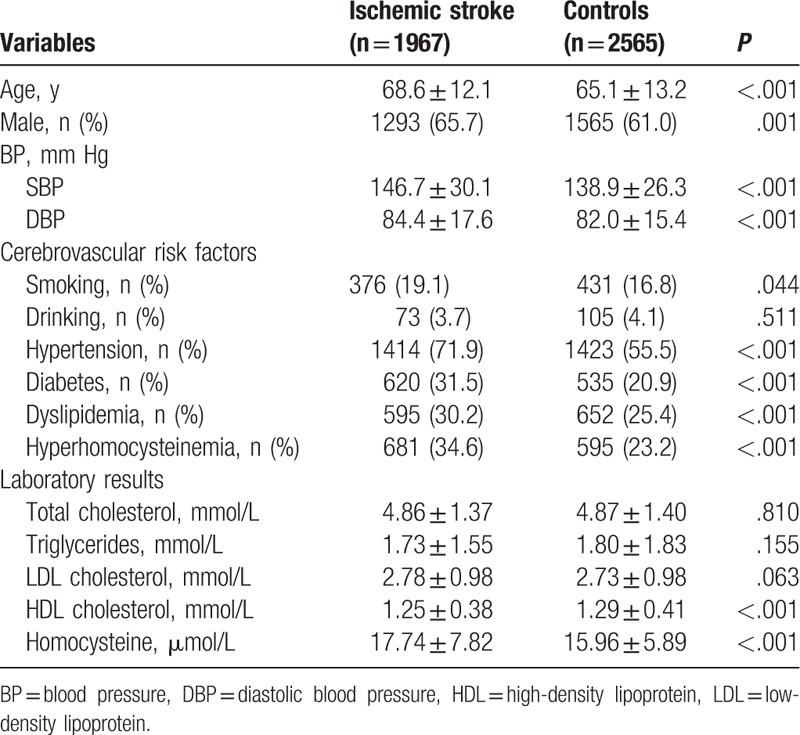
Baseline clinical characteristics of ischemic stroke patients and controls.

**Table 2:**
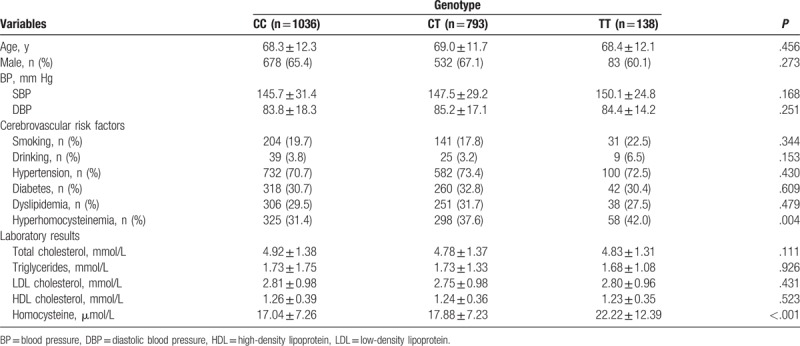
Baseline clinical characteristics of ischemic stroke patients according to *MTHFR* genotypes.

The genotypes distribution and allele frequencies of the studied *MTHFR* C677T polymorphisms in the ischemic stroke patients and controls are depicted in Table 3. Distribution of *MTHFR* C677T genotypes in the ischemic stroke patients (*χ*^2^ = 0.681, *P* = .409) and in the controls (*χ*^2^ = 1.507, *P* = .220) were consistent with the HWE expectations. Frequencies of CC, CT, and TT genotypes were 52.67% versus 55.63%, 40.31% versus 38.52%, and 7.02% versus 5.85% in patients with ischemic stroke versus controls, respectively. Among which, the homozygous TT genotype (7.02%) in the ischemic stroke patients was associated with increased risk (*P* = .049, OR 1.132, 95% CI 1.001–1.281) when compared with the controls (5.85%) after adjustment for age and sex. Also, dominant (TT + CT vs CC) and recessive models (TT vs CT + CC) were also performed to assess the effect of *MTHFR* C677T on the risk of ischemic stroke. The positive association was only found in dominant model without adjustment for age and sex (*P* = .047, OR 1.127, 95% CI 1.002–1.268). The T-allele frequency of *MTHFR* C677T was significantly higher in ischemic stroke patients (27.17%) than in controls (25.11%) (*P* = .026, OR 1.113, 95% CI 1.013–1.223).

**Table 3:**
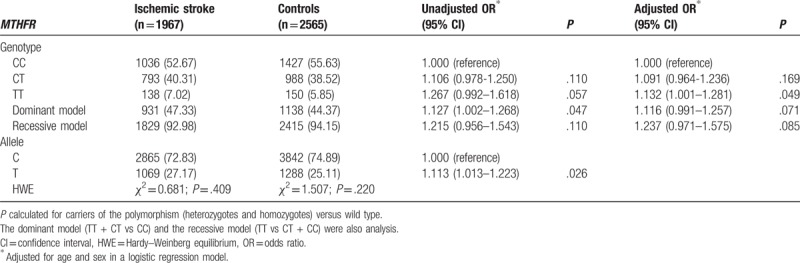
Genotypes and allele frequencies of the *MTHFR* C677T polymorphisms in ischemic stroke patients and controls.

Multivariate logistic regressions were used to examine the risk factors for ischemic stroke. As shown in Table 4, age, hypertension, diabetes mellitus, dyslipidemia, and hyperhomocysteinemia, but not sex, was found to be significant risk factors for ischemic stroke (*P* < .01). Moreover, the carrier status of the *MTHFR* T allele was identified as an independent risk factor for ischemic stroke even after the adjustment for conventional risk factors (*P* = .047, OR 1.109, 95% CI 0.964–1.225).

**Table 4:**
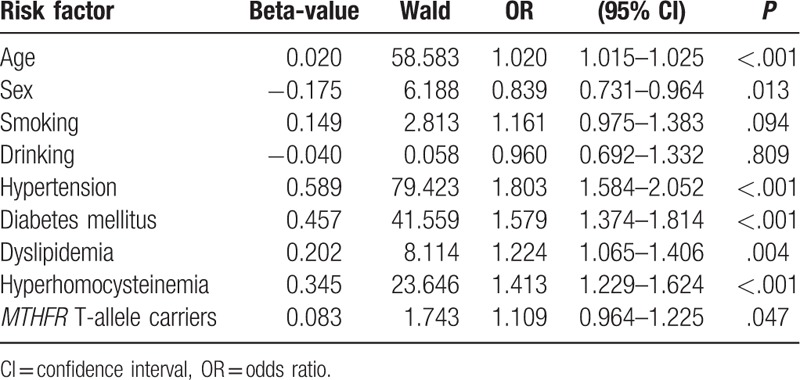
Multiple logistic regression analysis using ischemic stroke as the dependent variable.

## 4 Discussion

The mortality and morbidity of ischemic stroke remain very high in different districts. Ischemic stroke is a complex multifactorial disorder, resulting from the interaction by several genetic and environmental factors.^[4,8]^ Previous study has shown that the *MTHFR* C677T polymorphisms were associated with ischemic stroke. It has also been proposed that known genetic abnormality associated with ischemic stroke may not be the same in individuals with different genetic backgrounds.^[20,22]^ Till now, little is known about the association of *MTHFR* C677T polymorphisms and the risk of ischemic stroke in the Chinese Hakka population. Therefore, in the present study, we have detected such association in a southern Chinese Hakka population. In our study population, the homozygous TT genotype significantly increases the risk of ischemic stroke after adjustment for age and sex (*P* = .049, OR 1.132, 95% CI: 1.001–1.281). The positive association was found in dominant model without adjustment for age and sex (*P* = .047, OR 1.127, 95% CI 1.002–1.268). We also showed a significantly higher frequency of T allele in ischemic stroke patients (*P* = .026, OR 1.113, 95% CI 1.013–1.223). In addition, other factors, including the presence of age, hypertension, diabetes mellitus, dyslipidemia, and hyperhomocysteinemia, were associated with the risk of ischemic stroke (*P* *<* .01). Meanwhile, the carrier status of the *MTHFR* T allele was identified as an independent risk factor for ischemic stroke even after the adjustment for conventional risk factors (*P* = .047, OR 1.109, 95% CI 0.964–1.225).

The prevalence of the mutations of *MTHFR* C677T varies substantially different in distinct geographical region, races, and the ethnicity populations. For example, the frequency of T allele was reported to be high in European, North Americans, and East Asians, but low in African populations.^[23–26]^ Interestingly, a north-to-south cline of increase in allele frequency has also been observed in Europe, but a reverse trend geographical gradients among Chinese populations.^[27–29]^ In our study, the frequencies of CC, CT, and TT genotypes were 52.67%, 40.31%, and 7.02% in ischemic stroke patients, and 55.63%, 38.52%, and 5.85% in the controls, respectively. The frequency of T allele was 27.17% in ischemic stroke patients and 25.11% in the controls, respectively. The individuals included in the study were at least 3 of their grandparents originated from Meizhou region, which means that the population we studied was representative. Our results indicated that the genotype distribution and allele frequencies of the *MTHFR* C677T polymorphisms in our population differ from that of other regions and ethnicities.

Moderate elevation of plasma total homocysteine has been shown to be associated with ischemic stroke. *MTHFR* is a regulatory enzyme of the homocysteine metabolism, and the most common *MTHFR* C677T polymorphisms has been reported to be a strong predictor of hyperhomocysteinaemia; the study suggested that a causal relationship might be accounted for by a lower enzyme activity in the subjects possessing the *MTHFR* TT genotype.^[16,17]^ These observations have raised the possibility of a correlation between *MTHFR* C677T polymorphisms and the risk of ischemic stroke. However, the results were inconsistent, and very often even conflicting. The elevated plasma homocysteine levels were associated with the presence of *MTHFR* C677T polymorphisms, which may be associated with an increased risk of ischemic stroke among Singapore, Malaysian, Tunisian, Chinese, and Polish populations.^[30–33]^ However, no relation between *MTHFR* C677T polymorphisms and the risk of ischemic stroke has been observed in Turkey, Zambian, and Brazilian populations.^[34–36]^ Even for the people in the same country, for instance Indian, there was a dramatically different susceptibility to ischemic stroke, showing both decreased and increased risk.^[37–39]^ In the Turkish population, the same inconsistency could also be observed.^[40,41]^ Although the discrepancy between the results from all those studies is unclear and may be due to variation in sample size, ethnic differences and nutritional intake of cofactors were required for the *MTHFR* pathway, such as folate or vitamin B12.^[42,43]^ Our research revealed that the T-variant carriers of *MTHFR* C677T were significantly associated with a higher susceptibility to ischemic stroke (Table 3). Meanwhile, the combination of TT genotype with CT genotype appeared to increase the risk of ischemic stroke without adjustment for age and sex. Furthermore, the homozygous TT genotype was found to significantly increase the risk of ischemic stroke after adjustment for age and sex. These are consistent with the most previous studies, and we postulate that the genetic risk variants of *MTHFR* C677T might affect ischemic stroke risk by jointly modulating homocysteine levels, but further studies are needed to uncover the precise mechanism behind the regulation of *MTHFR* C677T polymorphisms in ischemic stroke.

It has been generally accepted that both lifestyle and genetic make-up influence the genetic susceptibility to ischemic stroke. Many of the traditional risk factors are well known to be associated with the increased risk of ischemic strokes, such as hypertension, diabetes mellitus, and cigarette smoking, and are modifiable or avoidable.^[8,18]^ In this study, by multivariate logistic regressions analysis, we found that age, hypertension, diabetes mellitus, dyslipidemia, and hyperhomocysteinemia were statistically significant risk factors for ischemic stroke, whereas sex was served as a protective factor. Hakka is an intriguing Han population that mainly inhabits in southern China, but with northern Han cultural traditions and linguistic influences.^[44]^ It has been proposed that Hakka population living in Meizhou area purely originated from Central Plain—a vast region containing the current Shanxi and Henan Provinces.^[45,46]^ The Hakka population is characteristic of their unique culture including some features in dialects, life styles, customs, and habits. For instance, the famous architectural type of Hakka—Round-Dragon House—is suggested to be derived from northern Han courtyard house. Also, the Hakka population is fond of foods high in saturated fat and sodium such as preserved meat. These diet habits may contribute to the development of a series of functional disorder such as hypertension, dyslipidemia, and hyperhomocysteinemia in the Hakka population, and eventually lead to ischemic stroke. In this study, our data also confirm that homozygous T carriers possessed higher homocysteine plasma levels. Moreover, the carrier status of the *MTHFR* T allele was identified as a moderate risk factor for ischemic stroke, even after the adjustment for traditional risk factors. These findings suggest that the possible effects of *MTHFR* C677T mutation on ischemic stroke may mediate through elevated homocysteine plasma levels in the study population.

Our study has several advantages. Our population was enrolled from Meizhou, which is a small town located in the northeast of Guangdong Province. The region has a high geographic stability, which could significantly reduce the potential confounding effects of the heterogeneous participants in the study. However, some limitations of this study should be pointed out. First, there may be differences in the results of patients from different regions and races. Therefore, these results need to be verified in other populations. Second, the present study is limited by the fact that the lack of supplement information on dietary folate intake and plasma folate concentrations was not evaluated, and that the effects of *MTHFR* genotype may be modulated by folate status. Third, we chose to study only the polymorphisms of *MTHFR* C677T genes. Because stroke is a complex disease, there is a lack of conviction to study only 1 gene in individuals. A cumulative effect of multiple genotypes and an interaction between specific genetic and environmental may contribute to the emergence of an ischemic stroke event.

## 5 Conclusions

In summary, we provide evidence that homozygous TT genotype carriers of *MTHFR* had significantly higher plasma homocysteine levels and increased risk factor for ischemic stroke in the southern Chinese Hakka population. Given the hypothesis that *MTHFR* C677T mutation is a risk factor for ischemic stroke, identification of high-risk populations with genetic predisposition to ischemic stroke will promote the development of strategies for prevention and treatment of ischemic stroke in our study population.

## Acknowledgments

The author would like to thank other colleagues whom were not listed in the authorship of Center for Cardiovascular Diseases, Clinical Core Laboratory and Center for Precision Medicine, Meizhou People's Hospital (Huangtang Hospital), Meizhou Hospital Affiliated to Sun Yat-sen University for their helpful comments on the manuscript.

## Author contributions

Contributions: Pingsen Zhao conceived and designed the experiments; Jingyuna Hou recruited subjects and collected clinical data and conducted the laboratory testing. Xing Zeng, Yunquan Xie and Heming Wu helped to analyze the data. Pingsen Zhao and Jingyuna Hou prepare the manuscript. Pingsen Zhao reviewed the manuscript.

**Conceptualization:** Pingsen Zhao.

**Data curation:** Jingyuan Hou, Yunquan Xie, Hesen Wu, Pingsen Zhao.

**Formal analysis:** Pingsen Zhao.

**Funding acquisition:** Jingyuan Hou, Pingsen Zhao.

**Investigation:** Jingyuan Hou, Pingsen Zhao.

**Methodology:** Jingyuan Hou, Xing Zeng, Yunquan Xie, Hesen Wu, Pingsen Zhao.

**Project administration:** Pingsen Zhao.

**Resources:** Jingyuan Hou, Xing Zeng, Yunquan Xie, Hesen Wu, Pingsen Zhao.

**Software:** Jingyuan Hou, Xing Zeng, Yunquan Xie, Hesen Wu, Pingsen Zhao.

**Supervision:** Pingsen Zhao.

**Validation:** Pingsen Zhao.

**Visualization:** Pingsen Zhao.

**Writing – original draft:** Jingyuan Hou, Pingsen Zhao.

**Writing – review & editing:** Pingsen Zhao.
